# Characterization of a polyclonal antibody that is highly selective for the d-isoAsp-25 variant of mammalian histone H2B

**DOI:** 10.1007/s00726-023-03242-z

**Published:** 2023-01-30

**Authors:** Dana W. Aswad, Kevin S. O’Leary, Katherine Williams

**Affiliations:** 1 Department of Molecular Biology and Biochemistry, University of California, Irvine, CA 92697-3900 USA; 2 Department of Developmental and Cell Biology, University of California, Irvine, CA 92697-2300 USA

**Keywords:** d-Amino acids, Histones, Gene expression, Chromatin

## Abstract

Approximately 12% of histone H2B molecules in mammalian brain contain a modification wherein Asp25 is present as the d-enantiomer, and is mostly linked to Gly26 via the side-chain carboxyl. Here we (1) demonstrate the high specificity of a polyclonal antibody to this modification, and (2) use this Ab to demonstrate that this modification is enriched in brain relative to liver, thymus, and HeLa cells.

## Introduction

The enzyme protein l-isoaspartyl methyltransferase (PIMT) selectively methylates the free α-carboxyl of atypical l-isoAsp sites in damaged proteins as the first step in converting the isopeptide bond into a normal peptide bond. The succinimide intermediate that forms during this repair reaction is susceptible to racemization, leading to small amounts of d-isoAsp (and presumably some d-Asp) as significant by products (Johnson et al.^1987^; McFadden and Clarke 1987).

The phenotype of PIMT −/− (KO) mice is dominated by neurological dysfunction leading to fatal epileptic seizures at 4–6 weeks (Kim et al. 1997; Ikegaya et al. 2001). These mice made it possible to identify proteins that are highly susceptible to isoAsp formation in a proteomic study of brain cytosol that revealed over 30 such proteins, many of which play key roles in neuronal function (Zhu et al. 2006). A previous search for PIMT targets in the nuclear fraction of KO mouse brain revealed only one such protein, a novel variant of histone H2B that harbors an l-isoAsp site at Asp25-Gly26 in the N-terminal domain (Young et al. 2001). Given the relatively long half-life of histones, we speculated that repeated cycles of damage and repair to H2B in PIMT + / + (WT) mice might lead to significant accumulation of d-isoAsp/d-Asp at this site. Indeed, we found Asp25 is present as the d-enantiomer in approximately 12% of histone H2B molecules isolated from adult mouse or dog brain (Young et al. 2005).

A polyclonal antibody made against a synthetic peptide corresponding to amino acids 21–31 of H2B, with D-isoAsp at position 25, provided preliminary evidence for its enrichment in active vs repressed chromatin (Qin et al. 2016). With the intent of using this antibody to obtain more information about its potential significance in gene regulation, we present here a more detailed characterization of this antibody per recommendations of the ENCODE consortia (Landt et al. 2012). Information on the relative levels of this H2B modification in several mammalian tissues is also presented.

## Materials and methods

Human recombinant H2B (M2505S) was obtained from New England BioLabs (Ipswich, MA). Calf thymus histones (LS002544) were from Worthington Biochemical Corp. (Lakewood, NJ). Mouse histones (brain and liver) and nuclear extract (brain) were prepared as previously described (Young et al. 2001). HeLa cell histones (16-0002) and nuclear extract (17-0001) were purchased from EpiCypher (Durham, NC).

The H2B V119 loading-control antibody (8135) was from Cell Signaling Technology (Danvers, MA). Our custom antibody to the D-isoAsp-H2B peptide was described previously (Qin et al. 2016). HRP-conjugated secondary antibody (donkey anti-rabbit; NA934) was from GE Healthcare.

Proteins were separated by SDS-PAGE on 16% Novex Tris–Glycine gels and transferred to nitrocellulose (Invitrogen transfer stack PB3210) for 11 min at 0.5 Amp per gel using a Thermo-Fisher Power Blotter XL. Membranes were blocked for 1 h in 5% BSA then incubated for 1.5 h in primary antibody, followed by 45 min in secondary antibody. Blocking and antibody solutions all contained 1X TBST. Membranes were washed 4X for 5 min in TBST after each antibody incubation. Imaging was carried out using Thermo-Pierce ECL2 Western Substrate (80196) per the included instructions, and images were captured on a Nikon D700 SLR camera (Khoury et al. 2010).

## Results and discussion

**Fig. 1:**
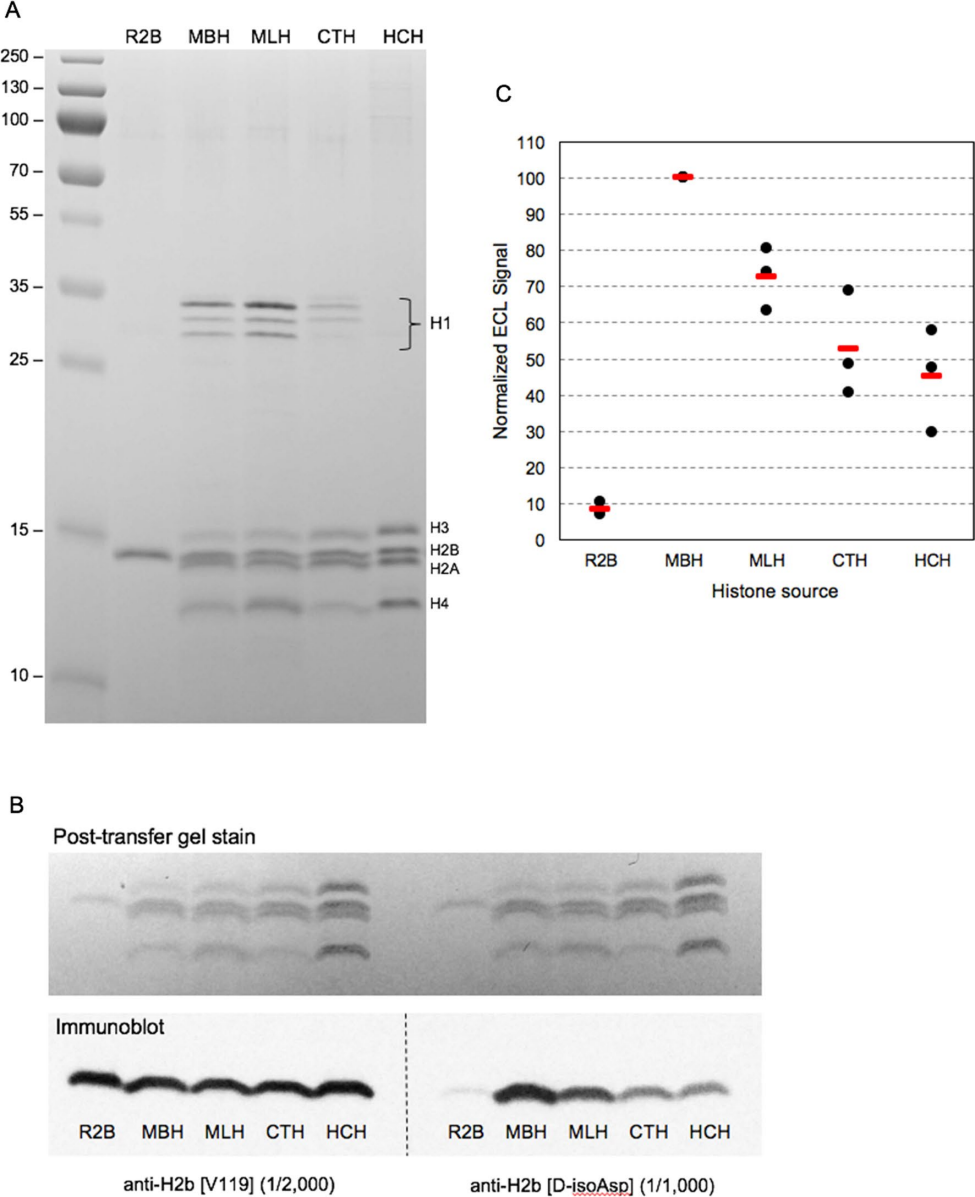
Specificity of the H2B d-isoAsp antibody against histones from several sources. See text for details

We used Western blotting to compare the specificity of the anti-D-isoAsp-H2B antibody against histones isolated from mouse brain (MBH), mouse liver (MLH), calf thymus (CTH), Hela cells (HCH), and recombinant human H2B (R2B) as a modification-free control. Figure 1A shows a stained gel demonstrating the composition and purity of the histone preps used in this study. Figure 1B (lower panel) shows immunoblots of the histones using our anti-D-isoAsp-H2B antibody (right series), and a commercial loading-control antibody (left series) made against a synthetic peptide encompassing V119 in the C-terminal region of H2B. The upper panel of Fig. 1B is a post-transfer gel stain used to verify that transfer efficiency was the same on both sides of the gel used in this experiment. The data shown here are representatives of three such blots. The results shown in Fig. 1B demonstrate that our antibody reacts strongly with naturally occurring H2B, very poorly with recombinant H2B, and not at all with other histones. A quantitative assessment of the d-isoAsp antibody specificity is shown in Fig. 1C, employing corrections for the small differences in sample loading. Immunoreactivity against MBH was found to be 10 times that of R2B. We know however, from HPLC data (Young et al. 2005), that only 12% of H2B molecules in MBH contain a D-Asp. So on a mol/mol basis, our antibody has a specificity of 10/0.12 = 83-fold compared to R2B. Because the -Asp-Gly- sequence in H2B is inherently susceptible to spontaneous succinimide formation, it is possible that the weak R2B signal is due in part to trace amounts of d-isoAsp.

**Fig. 2:**
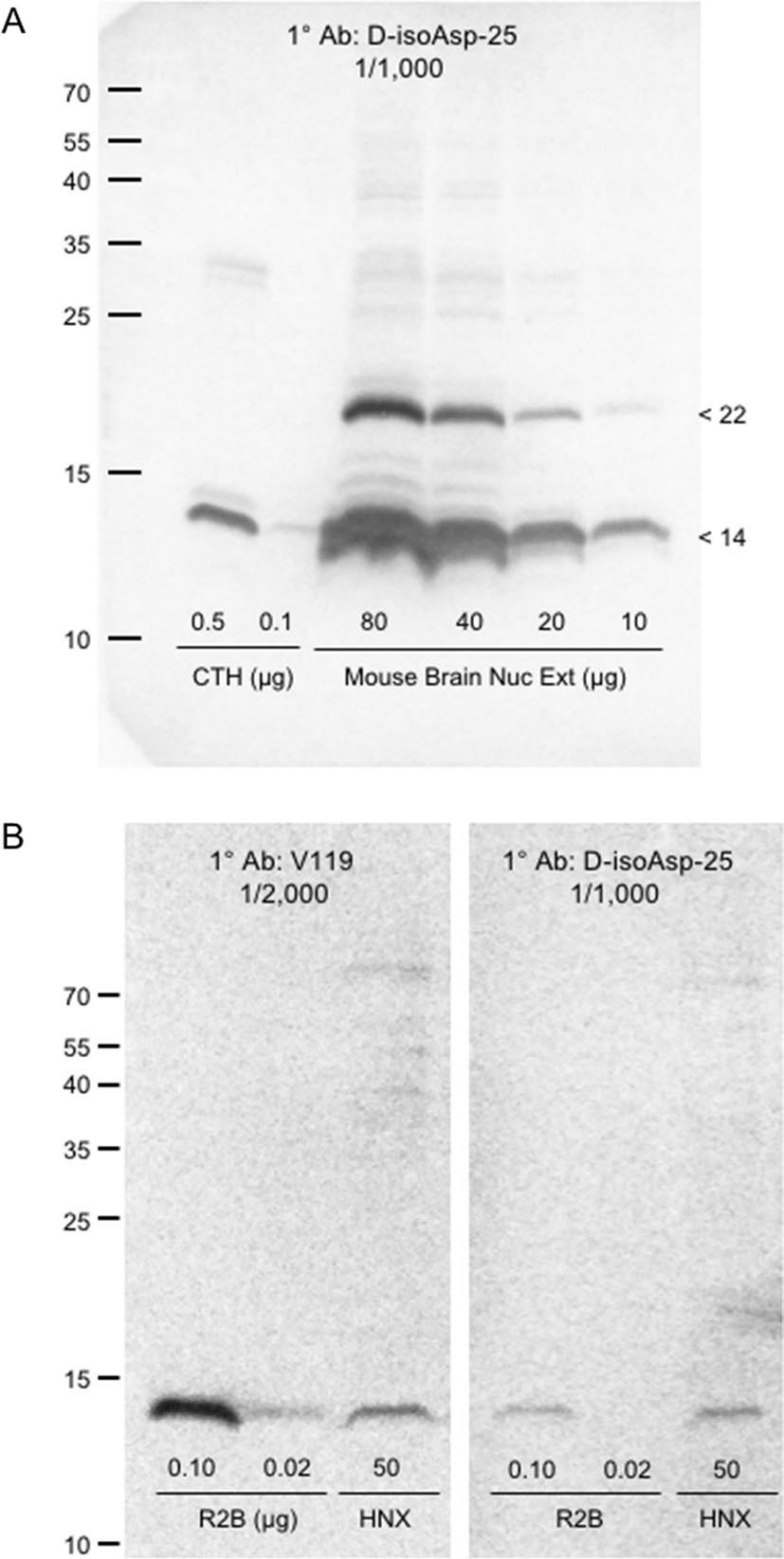
Evidence that histone H2B is the major target for the d-isoAsp antibody in nuclear extracts. See text for details

In Fig. 2, we explored the specificity of our d-isoAsp H2B antibody using Western blots of nuclear extracts from mouse brain (2A) and HeLa cells (2B). Figure 2A shows evidence of two bands in the mouse brain nuclear extract that react with our antibody. The major band (at 14 kDa) co-migrates with the H2B in CTH, while the minor band migrates 8–9 kDa higher. Ubiquitin has a mass of 8.6 kDa and is known to be a modifier of H2B in vivo (Emre and Berger 2004). The left panel of Fig. 2B (using the V119 antibody) shows that our commercially sourced nuclear extract of HeLa cells (HNX) does contain histone H2B, albeit at surprising low levels. (The results in this panel were replicated with another lot of extract from the same supplier.) The right panel (using our d-isoAsp antibody) shows reactivity in HNX only in the position of H2B. Because of the low levels of histone in this nuclear extract, it is not possible to rule out other minor reactive bands such as the 22 kDa band seen above in 2A.

We believe the results reported here meet the Primary ENCODE test requirements for ChIP-based studies on histone modification (Landt et al. 2012). These include (1) “…the specific histone band should constitute at least 50% of the signal in Western blots of nuclear extract…”, (2) “… show at least tenfold enrichment relative to any other single band” and (3) “…show at least tenfold enriched signal relative to unmodified histone”. In meeting one of the listed secondary tests (“Mutants defective in modifying histones”.), we have previously shown that our d-isoAsp antibody elicits a greatly reduced Western blot signal against histones from PIMT KO mice vs. WT mice (Qin et al. 2016).

In H2B of mammals and yeast, the succinimide-prone -DG- bond immediately precedes a highly basic patch of eight amino acids known as the histone 2B repression domain (HBR) which has been implicated in regulating gene expression and DNA repair (Parra et al. 2006; Zheng et al. 2014; Rodriguez et al. 2018). The H2B D-isoAsp modification is thus in a position to dramatically affect how the N-terminal tail of H2B interacts with other histones, nucleosomal DNA, and transcription factors. ChIP-seq type studies using the antibody characterized here should prove useful in exploring possible functions of this highly unusual histone modification. We have not tested the efficacy of this antibody against aldehyde-treated (cross-linked) chromatin (xChIP), but cross-linking is not generally needed for ChIP studies related to covalent histone modification due to the strong interaction between histones and DNA. Any laboratory that would like to make use of this antibody is encouraged to contact Prof. Dana Aswad at the email address provided.

## Data Availability

The data used in this work are available upon request via email to dwaswad@uci.edu.
